# Institutional Notes and News

**Published:** 1911-02-18

**Authors:** 


### Institutional Notes and News.

An increased subscription list has improved the good fortunes of the David Lewis Northern Hospital, Liverpool. "With the amount transferred from the legacies account, the nursing institution account, the ambulance fund, and the increase in donations and subscriptions, the hospital has been more than able to pay its way." The in-patients numbered 2,467, there were 17,878 out-patients, and the ambulance was called 923 times. The total expenditure was £11,383, and the income, which included £1,500 transferred from the legacies account, was £16,846. The debt on the current account is now £5,581, and outstanding debts are £1,053. A moral from these figures was drawn by the Lord Mayor, who said that "it was a wise plan to use for current expenses those legacies which were not accompanied by specific directions for their use." This contention he illustrated by saying that the London hospitals were in a different position, as they often drew large incomes from previous investments in land. This however, he thought, made them appeal less to public charity.

**Northern Hospital, Liverpool.**

Mr. P. W. Kersley, chairman of the Board of Management, presided over the annual meeting of the Manchester Royal Eye Hospital. It was then admitted regretfully that the money subscribed towards the new buildings had been insufficient to pay for them, and, as the hospital's friends may remember, the trustees' sanction had been resorted to so that legacies were employed for this end. This policy is to continue during the present year, and it is pointed out to the public that a debt of £1,000 is threatened if no fresh effort is made to subscribe sufficient to allow the investment of legacies. The increased number of in-patients justifies the increased accommodation, 352 more, or a total of 1987, in-patients having been received. Mr. Kersley, however, remarked : "There is room for 30 more beds, and these beds could be equipped and filled if we had the money." This sums up the present condition of the hospital, and its friends should be roused by this to make increased efforts on its behalf.

**Manchester Royal Eye Hospital.**

The Brixham Cottage Hospital has had a busy year, and one of the most healthy signs of its condition is the large number of subscribers and supporters which attended the annual meeting last week. Sometimes, however much it may be disguised, the annual meeting of a cottage hospital is not regarded by the neighbourhood as a function which it is worth while to attend. This is generally due to the extreme dullness of the speakers. The gentleman who presided on the present occasion was Mr. W. J. Sanders, who is chairman of the General Committee, and he managed to make the report and balance sheet digestible. The report of a cottage hospital is only as dull as its compilers choose to make it, and we are glad to note the tendency of the past few years to insist that something shall be done to make reports more readable. There were 90 in-patients, 191 general district, and 109 maternity cases—an extensive list which shows that the Brixham Hospital is appreciated. The income has nearly doubled in twelve months, or, if strict accuracy must demand the admission that some stock was sold, increased by one-third. The proposed institution of a Hospital Sunday in August is only one sign that the Brixham Cottage Hospital's committee is alert and progressive.

**Brixham Cottage Hospital.**

The old Rotunda at Dublin was the scene last week of a successful ball in aid of the Mercer's Hospital. This, we believe, is actually the oldest hospital in Dublin, and the dance on its behalf attracted all young Dublin. Probably the Mercer's Institution is the most popular of its kind in the city, and its long association in the minds of the citizens with the treatment of many generations of them accounts for this. When an institution can preserve the prestige of an old tradition without sacrificing its buildings, or other ties to the past, to modern science or craziness, it has a very rare combination of virtues. We hope that the Coronation year, which is to include a Royal visit to Ireland, will not be allowed to pass without special efforts having been made to bring every Dublin hospital up to the standard which was first set in the new reign by King George's inspection of the London Hospital.

**Mercer's Hospital, Dublin.**

A Record year was the subject of discussion at the Annual Court of Governors of the Sunderland Infirmary last week, when Mr. Alderman Richardson presided. The total number of patients treated has risen to 16,066 for the year, one-fifth of whom were in-patients, while the institution is naturally proud of the fact that the 606 additional persons who have received treatment has not sent up the expenditure. It is true, however, that this result has been gained partly by deferring to a future time certain extraordinary expenses in connection with the administration block. These can not be put off much longer, and there should be little need to postpone them as the Hospital's annual subscriptions have shown a useful increase, the miners especially having made very generous efforts on behalf of their Infirmary. Two other points deserve notice, the lesser of the two being dismissed first—namely, the need for a Children's Hospital for which £5,000 is wanted. It will cost an additional £2,000 a year, and will be opened when there is a reasonable chance of this sum being received. The other matter is the petition of the Sunderland Infirmary to the King praying him to grant to them the title of Royal, with which we deal in another column. Dr. G. B. Morgan deserves the public's thanks and support for having called the meeting's attention to the want of any proper accommodation for diphtheria cases. There is, we understand, no isolation hospital or ward in the district.

**Sunderland Infirmary.**

Only last week we had the pleasure of recording a generous present to the Moreton Hampstead Cottage Hospital from its president the Hon. W. F. D. Smith. Since then he has set the seal on his chairmanship of the King's College Hospital Removal Fund by his promise to pay the cost of one of the central three-storeyed ward blocks of the new hospital. The cost of a block is expected to be £30,000, or, in other words, one-third of the total amount asked for by the building-fund section of the Appeal Committee. There will be another block similar to the one which Mr. Smith's abundance has provided, and these, with the administration buildings, are to form the new hospital. We must not forget to add that the administration buildings will have a low wing on either side, one of which will accommodate the Medical School and the other will accommodate the out-patients. This last is being built rapidly, and the casualty section will be finished soon. We hope that Mr. Smith's great example will lead to emulation, and that the Appeal Committee will shortly have concluded its work.

**King's College Hospital.**

